# Subacute brainstem ischemic syndrome in juvenile neurofibromatosis type 2: An under‐recognized condition

**DOI:** 10.1002/ccr3.6804

**Published:** 2023-01-03

**Authors:** Aglaë Blauen, Christine Lenfant, Thierry Duprez, Marie‐Cécile Nassogne

**Affiliations:** ^1^ Department of Child Neurology, Cliniques Universitaires Saint‐Luc Université Catholique de Louvain Brussels Belgium; ^2^ Department of Radiology, Cliniques Universitaires Saint‐Luc Université Catholique de Louvain Brussels Belgium

**Keywords:** brainstem ischemic syndrome, locked‐in syndrome and type 2 neurofibromatosis

## Abstract

We report the case of a teenager with neurofibromatosis type 2 (NF2) presenting a locked‐in syndrome due to a brainstem ischemic syndrome. The presence of sudden or rapidly worsening onset of neurological deficits in NF2 patients should evoke this underknown entity and not only tumors as predisposed by NF2.

## 1 INTRODUCTION

Neurofibromatosis type 2 (NF2) is an autosomal‐dominant tumor‐prone disorder characterized by the development of distinctive nervous system tumors, including meningiomas, ependymomas, and peripheral, spinal, and cranial nerve schwannomas, in addition to skin anomalies and visual symptoms. Bilateral vestibular schwannomas are pathognomonic. So, the most common entry into the disease is through hearing impairment, which usually occurs in the second decade of life. But, unlike adults, children most frequently present with ocular, dermatological, and neurological symptoms. Brainstem ischemic syndrome, which is an under‐recognized entity of unknown origin, occurs in teenagers without any previously known NF2 diagnosis, presenting as an acute or subacute event, which involves the midbrain or pons.

## 2 CASE HISTORY

A 15‐year‐old girl was transferred from a primary care institution for headache, acute left hemiparesis, and left paresthesia. Her history revealed left hypoacusis, left voice cord palsy, and left unreactive mydriasis of unknown origin for over a 3‐year period. The admission brain CT scanner had shown heterogeneous parenchymal damage within the right side of the pons (not shown). Three years earlier, the girl underwent brain magnetic resonance imaging (MRI), which was described as normal.

**FIGURE 1:**
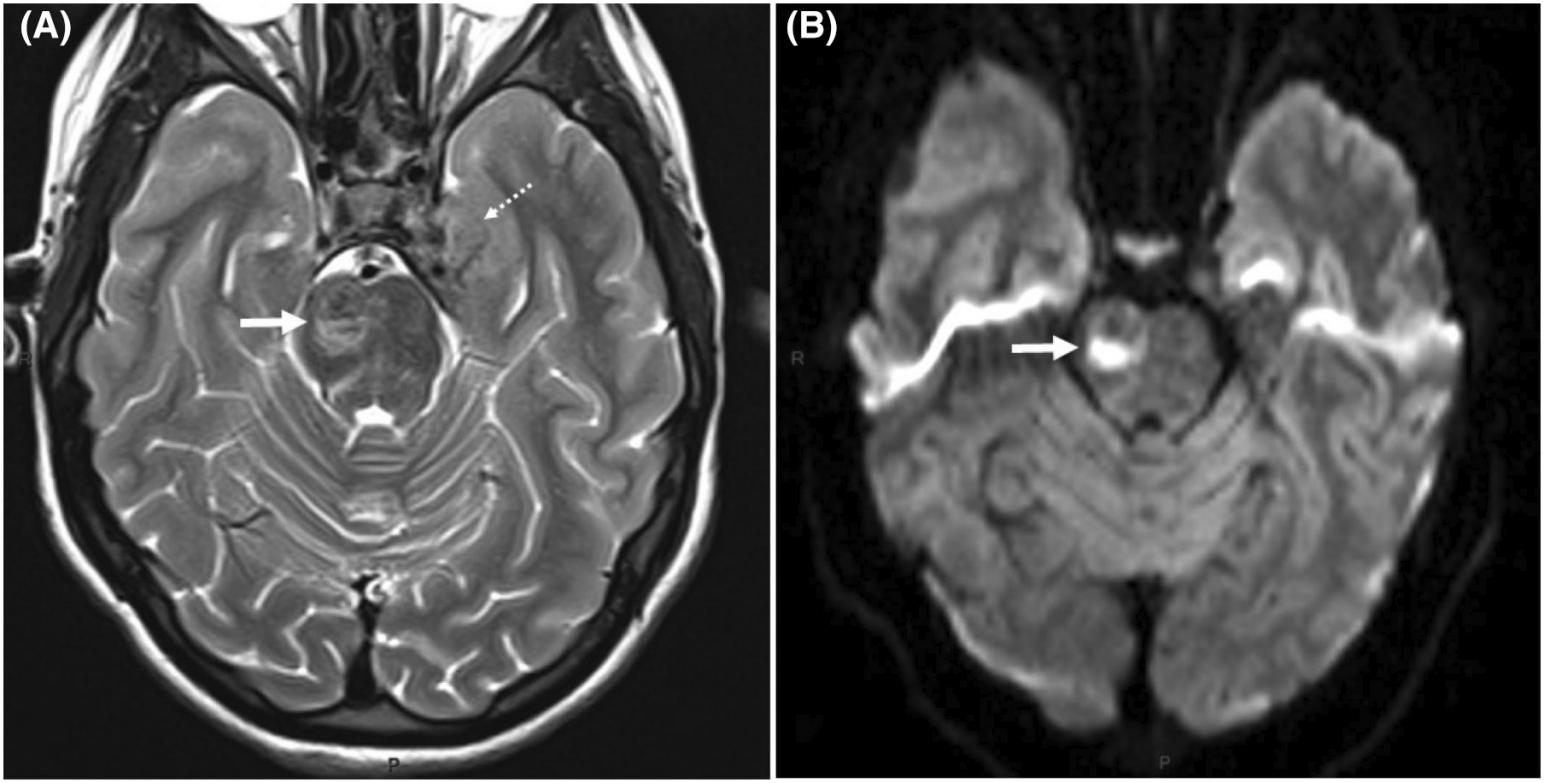
MRI work‐up at admission. Two axial transverse slices in similar slice location. (A) T2‐weighted (T2‐W) view shows heterogeneous parenchymal damage (arrow) of the right side of the ponto‐mesencephalic junction displaying with high signal intensity of its lower half which contrasts with low signal of its upper half. Partial volume on a schwannoma of the left Vth cranial nerve (dotted arrow). (B) DW view confirms the duality of the low/high signal intensities within the lesion (arrow) with high signal intensity of the lower half featuring cytotoxic edema.

The brain MRI at admission showed a heterogeneous T2/FLAIR hypersignal on the right thalami, cerebral peduncle, midbrain, and pons, displaying marked restriction of free water diffusivity featuring cytotoxic edema on diffusion‐weighted (DW) views. A few pontine subareas disclosed low signal intensity on both T2 and diffusion weightings suggesting foci of hemorrhagic transformation (Figure 1). Other multiple lesions already detectable on the MRI performed three years earlier, were present as bilateral vestibular schwannomas and left trigeminal schwannoma, together with a meningioma at the cranial vertex and a cervical cord ependymoma, strongly suggesting a type 2 neurofibromatosis (NF2).

**FIGURE 2:**
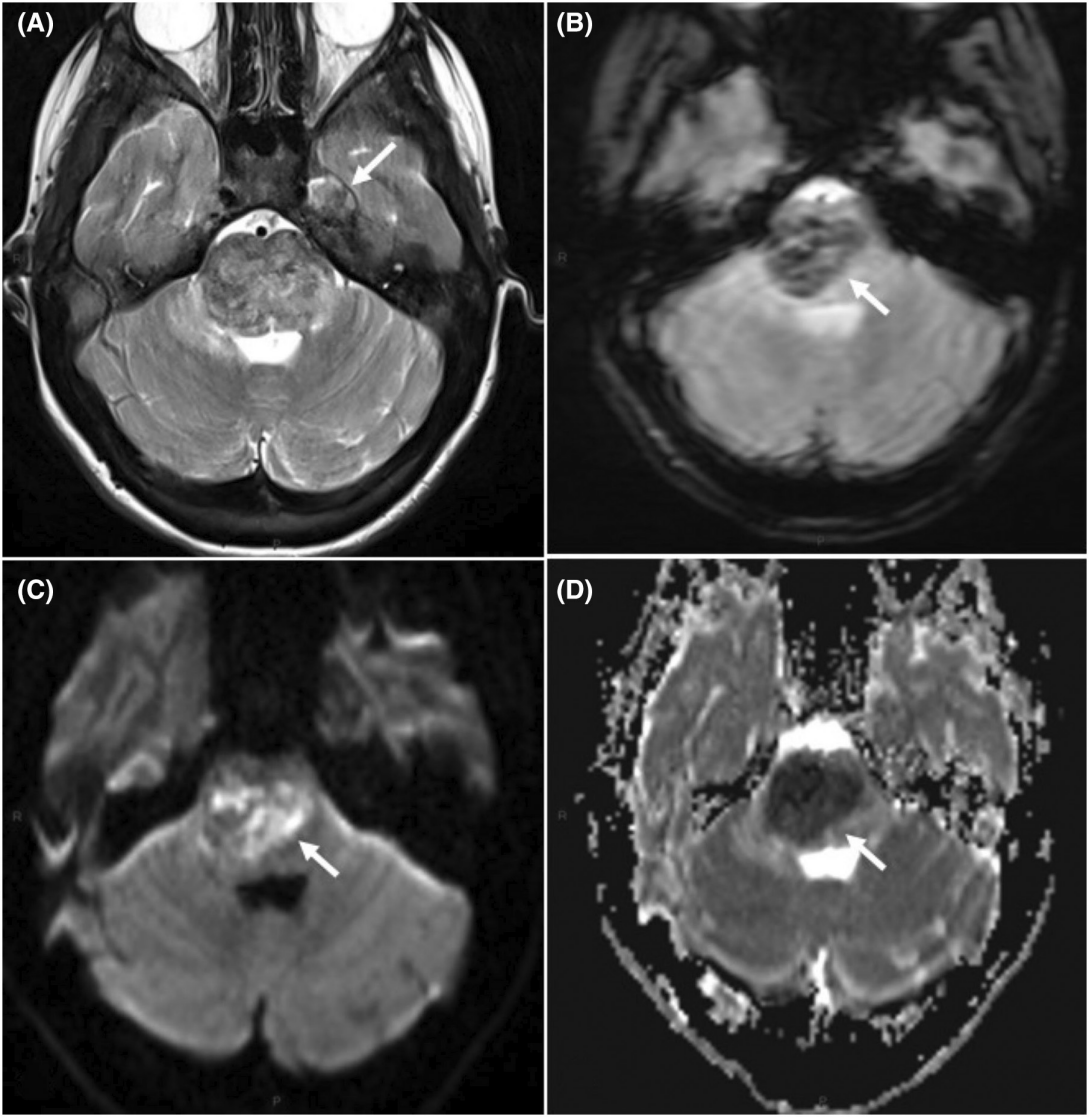
Follow‐up MRI two weeks later. Four axial transverse slices in similar slice location. (A) T2‐W view shows the extension of the parenchymal damage to the whole Pons with heterogeneous mosaicism of hyper/hypointense foci. Schwannoma of the left Vth cranial nerve (arrow). (B) gradient‐echo T2*‐W view shows strongly hypointense susceptibility artifacts revealing the presence of deoxyhemoglobin. (C) DW view shows hyperintense areas within the lesion (arrow) featuring cytotoxic edema due to ischemia. (D) ADC‐mapped view confirms a strong ischemia‐related decrease in apparent diffusion coefficient (ADC) values.

On Day 14, the left hemiparesis worsened, and the girl exhibited swallowing difficulties and bilateral facial palsy. The MRI, carried out on Day 18, revealed a progression of the right‐sided pontine lesion with persistent mosaicism of acute ischemic lesions with high T2/FLAIR signal intensity with lowered apparent diffusion coefficient (ADC) and strongly hypointense areas with susceptibility artifacts on gradient‐echo T2‐weighted views featuring hemorrhagic transformation (Figure 2).

From Day 20 onwards, the girl progressively developed spastic quadriplegia with pyramidal signs and a vesical globe; owing to her bilateral facial palsy, she was no longer able to speak, whereas her cognitive function was preserved. The diagnosis of a locked‐in syndrome was retained.

We reviewed the diagnostic differential of brainstem lesions (Table 1) and faced with this progressive lesion, a tumor was suspected, and corticosteroids were initiated. But stereotactic biopsy was not contributive to pathological changes evocating necrosis.

**TABLE 1:** Diagnostic differential of brainstem lesions

	Stroke	Tumor	Infection	Inflammation	Metabolic	Toxic
Etiology	Basilar artery occlusion (due to cardioembolism, trauma, hypercoagulable disorders, unknown) Vertebral artery dissection (+/− after neck traumatism) Brainstem ischemic syndrome in NF2 Unknown	Diffuse high‐grade glioma (+/− 75%) Focal low‐grade glioma Exophytic glioma (low‐grade) Langherans cell histiocytosis Epidermoid/dermoid tumor	*Listeria, Enterovirus, HSV*	ADEM Multiple sclerosis ANE (after *influenza A, B, parainfluenza, HHV6*) Bickerstaff encephalitis (ganglioside GQ1b antibodies, after *Campylobacter jejuni* or *Mycoplasma pneumoniae*)	Mitochondrial disease	Central pontine myelinolysis
Specific symptoms In addition to headaches, cranial nerve palsy, motor deficit	‐	Ataxia	Fever Encephalopathy Seizures Ataxia	+/− after a viral infection Encephalopathy Seizures Bickerstaff: Ophtalmoplegia, ataxia, coma, areflexia	+/− decompensation after a viral infection Multisystemic symptoms, ataxia, dystonia, regression	severe alteration of plasma osmolality or hyponatremia Encephalopathy Confusion
Brain MRI And other tests	T2 and flair hypersignal pons > medulla reduction in diffusion	T2 hypersignal High‐grade glioma: mass effect, oedema, infiltration, absent or inhomogeneous contrast enhancement Low‐grade glioma focal, less oedema Exophytic glioma from the 4th ventricle, as low‐grade glioma Langherans cell histiocytosis With hypothalamic–pituitary lesions (Epi)dermoid tumor Focal, no oedema, reduction in diffusion	T2 hypersignal multiple lesions, patchy, asymmetric, +/− abscess, +/− supratentorial lesions CSF: WBC ↑, protein normal or ↑, glucose: normal or ↓ Culture and PCR	T2 hypersignal with supra tentorial lesions in white matter (except Bickerstaff: only brainstem) CSF: WBC ↑, protein ↑, normal glucose, +/−oligoclonal bands, ganglioside GQ1b antibodies + in Bickerstaff	T2 hypersignal Gray matter: substantia nigra, medullary and pontine tegmentum, basal ganglia, and diffuse supratentorial leukoencephalopathy Blood test: lactate ↑, alanine ↑, urine organic acid: abnormal CSF: protein normal or ↑, lactate ↑ Molecular analysis	T2 hypersignal Central pontine myelinolysis central pons, +/− trident shape +/− midbrain and middle cerebellar peduncles Blood test: Osmolality alteration
Treatment	Anticoagulation or Aspirin +/− thrombolysis	According histology	Antimicrobial treatment	Corticoids	Vitamins and supportive therapy	Prevention Supportive therapy
Outcome	good except if size >50% or coma at presentation	According histology	Poor outcome in 50%	According etiology	Poor	Variable

Abbreviations: ADEM—acute disseminated encephalopathy, ANE—acute necrotizing encephalopathy, CSF—cerebrospinal fluid.

Then, by reviewing the girl's full medical record, the diagnosis of brainstem ischemic syndrome was proposed, reflecting an uncommon medical condition associated with NF2. Brain magnetic resonance angiography (MRA), cardiac‐carotid and vertebral ultrasonography, and hemostasis evaluation were unremarkable. Aspirin treatment was initiated, and she was transferred to a rehabilitation center. Three months later, she was able to carry out head movements, which helped her communicate, as well as some arm movements.

The NF2 diagnosis had meanwhile been confirmed by revealing a heterozygous pathogenic variant on *NF2* gene, c.1376dup (p.Glu460GlyfsTer35).

## 3 DISCUSSION

The locked‐in syndrome is a neurological disorder that is characterized by quadriplegia and anarthria with preserved cognitive functioning. Patients usually retain eye movements, thereby facilitating nonverbal communication. This clinical diagnosis may prove challenging, given that the children are often considered as being in coma or in an unresponsiveness state, or as displaying akinetic mutism. A normal electroencephalogram (EEG) should alert the physician.^1^ This condition is mostly caused by a brainstem lesion; in 61% of cases, the etiology in children is ventral pontine stroke due to vertebrobasilar artery thrombosis.^1^ The syndrome's prognosis depends on the underlying cause. Around 35% of patients experienced some motor recovery, 26% exhibited good recovery, 23% died, and 16% remained quadriplegic and anarthric.^1^ Motor recovery is earlier and superior in locked‐in syndrome nonvascular cases. Intensive rehabilitative care has been shown to improve motor outcomes.^1^

As illustrated by our case, differential diagnosis of brainstem lesions can be challenging. We reviewed the brainstem disorders in pediatric patients in an effort to guide the clinician in this difficult yet essential differential diagnosis. Usually, brainstem lesions tend to become quickly symptomatic. A small and single lesion can produce severe and mixed deficits related to the large number of essential structures localized within this area including cranial nerves nuclei, the reticular formation, and ascending, descending, and cerebellar pathways. Brain MRI is generally carried out early in the diagnostic approach. The resulting findings can help clinicians in regard to differential diagnosis.

Among the causes of brainstem pathologies in children, vascular, toxic, metabolic, infectious, inflammatory, or neoplasia processes appear to play a role, as previously reported. As degenerative diseases are rather rare in this specific area, they will not be discussed here. The clinical presentation of brainstem lesions is roughly uniform with multiple cranial nerve palsy, motor deficiencies, and headaches, whereas the clinical context, brain MRI features, and laboratory testing results may be quite useful in further directing the clinicians (Table 1).^2^

In our case, the diagnosis was difficult, given that the lesion progression led us to suspect the presence of a tumor, within the NF2 setting.

NF2 is an autosomal‐dominant disorder, caused by a variant inactivating the *NF2* gene encoding the protein merlin whose main function is to regulate cellular proliferation.^3^ Its loss of function is associated with several neurological tumors including peripheral, spinal, and cranial nerve schwannomas, meningiomas, as well as ependymomas, in addition to skin anomalies like NF2 skin plaques, subcutaneous, and cutaneous schwannomas, along with visual symptoms, including cataracts, retinal hamartomas, or optic nerve sheath meningiomas. Bilateral vestibular schwannomas in children, adolescents, and young adults are pathognomonic of the condition. The most common entry into the disease is through unilateral or bilateral hearing impairment, which usually occurs in the second decade of life. The presence of sudden or rapidly worsening onset of neurological deficits in NF2 patients should evoke a brainstem ischemic syndrome, which is an under‐recognized entity.^4^ This syndrome occurs in teenagers without any previously known NF2 diagnosis, presenting as an acute, usually monophasic event, which involves the midbrain or pons. A gradual evolution, as seen in our case, is similarly possible.^4^ In clinical terms, patients start to suffer from cranial nerve palsy, dysarthria, hemiparesis, or a locked‐in syndrome. The brain MRA, cardiac echocardiogram, and thrombophilia screen usually prove to be noncontributive. Several cases have been described as exhibiting vascular stenosis, which cannot explain the stroke in anatomical terms. So far, the etiology remains unclear. According to one of the hypotheses, the tumor suppressor protein merlin possibly plays a role in regulating physiological angiogenesis, whereas its inactivation may induce vascular dysplasia that could induce an ischemic event.^5^ Genetically, any distinct variant in *NF2* has been associated with brainstem ischemia. Another hypothesis raises the possibility of a digenic process, given that this syndrome is relatively rare in NF2 patients.^4^ Concerning treatment, aspirin can be employed, which is the case in ischemic events. The prognosis is variable. While some patients have fully recovered after 6 months, others did exhibit some motor recovery, and still, others remained in a locked‐in syndrome.^4^, ^6^ The evolution likely depends on both the ischemia extension and the quality of rehabilitative care.

## 4 CONCLUSION

Brainstem ischemic syndrome is a rare and under‐recognized entity of unknown origin, occurring in teenagers without any previously known NF2 diagnosis. The adolescents suffer from cranial nerve palsy, dysarthria, hemiparesis, or a locked‐in syndrome. The clinical context and MRI provide the diagnosis. So, it is essential to consider this syndrome in NF2 patients and not only tumors as predisposed by NF2. Aspirin can be employed. The prognosis is variable ranging from full remission to persistent locked‐in syndrome.

## AUTHOR CONTRIBUTIONS

AB contributed to the collection of the case information, reviewing the literature, designing, and writing the manuscript. CL contributed to the interpretation of brain MRI and was involved in drafting the manuscript. TD contributed to the interpretation of brain MRI, revised the manuscript critically for important intellectual content, and reshaped it. MCN contributed to the collection of the case information, reviewed the literature, established the diagnosis, revised the manuscript critically for important intellectual content, and reshaped it. All authors read and approved the final version of the manuscript.

## CONFLICT OF INTEREST

The authors have no conflicts of interest to declare.

## CONSENT

Written informed consent was obtained from the patient's parents to publish this report in accordance with the journal's patient consent policy.

## Data Availability

Data sharing is not applicable to this article as no new data were created or analyzed in this study.
